# Neutron activation increases activity of ruthenium-based complexes and induces cell death in glioma cells independent of p53 tumor suppressor gene

**DOI:** 10.1007/s10534-017-0006-1

**Published:** 2017-03-03

**Authors:** Aline Monezi Montel, Raquel Gouvêa dos Santos, Pryscila Rodrigues da Costa, Elisângela de Paula Silveira-Lacerda, Alzir Azevedo Batista, Wagner Gouvêa dos Santos

**Affiliations:** 1 Laboratório de Genética Humana e Biologia Molecular, Unidade Acadêmica de Ciências da Saúde, Regional Jataí, Universidade Federal de Goiás, cidade Universitária-Campus Jatobá, BR 364, Km 195, n. 3800, Jataí, CEP 75801020 Brazil; 2 Centro Nacional de Desenvolvimento da Tecnologia Nuclear, CDTN, Belo Horizonte, MG Brazil; 3 Laboratório de Genética Molecular e Citogenética, Instituto de Ciências Biológicas, Universidade Federal de Goiás - UFG, Goiânia, GO Brazil; 4 Departamento de Química, Universidade Federal de São Carlos, São Carlos, SP Brazil

**Keywords:** Glioblastoma, Brain tumor, Ruthenium(II), Metal complex, Radiotherapy

## Abstract

Novel metal complexes have received great attention in the last decades due to their potential anticancer activity. Notably, ruthenium-based complexes have emerged as good alternative to the currently used platinum-based drugs for cancer therapy, providing less toxicity and side effects to patients. Glioblastoma is an aggressive and invasive type of brain tumor and despite of advances is the field of neurooncology there is no effective treatment until now. Therefore, we sought to investigate the potential antiproliferative activity of phosphine-ruthenium-based complexes on human glioblastoma cell lines. Due to its octahedral structure as opposed to the square-planar geometry of platinum(II) compounds, ruthenium(II) complexes exhibit different structure–function relationship probably acting through a different mechanism from that of cisplatin beyond their ability to bind DNA. To better improve the pharmacological activity of metal complexes we hypothesized that neutron activation of ruthenium in the complexes would allow to decrease the effective concentration of the compound needed to kill tumor cells. Herein we report on the effect of unmodified and neutron activated phosphine ruthenium II complexes on glioblastoma cell lines carrying wild-type and mutated p53 tumor suppressor gene. Induction of apoptosis/authophagy as well as generation of reactive oxygen species were determined. The phosphine ruthenium II complexes tested were highly active against glioblastoma cell lines inducing cell death both through apoptosis and autophagy in a p53 independent fashion. Neutron activation of ruthenium compounds rendered them more active than their original counterparts suggesting a new strategy to improve the antitumor activity of these compounds.

## Introduction

Ruthenium has attracted much attention in the last decades as components of new transition-metal-based antitumor drugs (Clarke 2003; Zhang and Lippard 2003). Several studies have shown the reasons for the fast growing interest in ruthenium, as opposed to platinum, as component of metal-based complexes. Different ruthenium complexes with oxidation state 2^+^ or 3^+^ have demonstrated anticancer effects against a variety of cancer types and more importantly against metastatic cancers (Sava et al. 1999; Allardyce and Dyson 2001; Alessio et al. 2004; Nowak-Sliwinska et al. 2011).

The NAMI-A, imidazolium trans-[tetrachloro(dimethylsulfoxide)(1H-imidazole)ruthenate(III) underwent successfully phase I clinical trial on metastatic lung cancer (Sava et al. 2003), as well as KP1019, indazolium trans-[tetrachlorobis(1H-indazole)ruthenate(III) which showed activity against colorectal tumor model and several primary explanted human tumors (Berger et al. 1989). Similarly, ruthenium(II) arene complexes have shown potent antiangiogenic and antimetastatic effect (Clavel et al. 2015). Also, ruthenium/phosphine/diimine complexes have been synthesized and shown promising activity not only against cancer cells but bacteria as well (Dos Santos et al. 2013).

The specific target of antitumor ruthenium complexes remains to be elucidated, but it is generally accepted that ruthenium complexes cytotoxicity is related to their ability to bind DNA. However, several ruthenium compounds have also shown to inhibit DNA replication, induce SOS repair, reduce RNA synthesis and inhibit angiogenesis (Brabec and Novakova 2006; Nowak-Sliwinska et al. 2011). Uptake of ruthenium complexes by tumor cells has been suggested to occur through a mechanism mediated by the iron transport protein transferrin (Li and Qian 2002). Several studies have demonstrated an upregulation in expression of the transferrin receptor on metastatic and drug resistant tumours when compared to their normal counterparts (Ryschich et al. 2004; Sahoo et al. 2004; Sahoo and Labhasetwar 2005; Singh et al. 2011). Ruthenium is a transition metal of the same group as platinum and iron but shows important differences from platinum drugs, at the same time, it shares many positive characteristics with iron. Therefore it is possible that ruthenium mimics iron in its way to bind molecules of biological significance (Brabec and Novakova 2006). Furthermore, ruthenium complexes are thought to serve as prodrugs where activation-by-reduction and aquation are important steps related to the function of these complexes inside the cells (Clarke et al. 1999; Bacac et al. 2004; Jakupec et al. 2005). However, full understanding on the mechanism of action of ruthenium complexes still needs to be clarified.

Glioblastomas (GBM) are the most common brain tumors in adults and the most deadly primary tumors of the brain. It is characterized by its highly angiogenic potential and invasiveness to the surrounding brain tissue (Mangiola et al. 2013; Tanaka et al. 2013). GBM is also associated with very poor survival resistance to radiochemotherapy (Thuy et al. 2015). These characteristics continue to challenge researchers and physicians to find a successful strategy to treat patients diagnosed with this type of tumor. Therefore, the development of novel therapeutics agents able to inhibit the growth and kill glioblastoma cancer cells is urgent needed (Tanaka et al. 2013). Due to the demonstrated properties of ruthenium-based complexes and its broad range of activity against metastatic tumors, these metallodrugs may be useful candidates in the treatment of GBM tumors. Cisplatin based chemotherapy, one of the most used metallodrug for cancer therapy has shown limitations including: high toxicity, undesirable side effects and resistance. This fact motivates the investigation of new metal based cancer therapies. Although association of cisplatin to radiotherapy (RT) has improved prognosis for some cancers such as advanced cervical cancer, esophageal cancer and cancer of head and neck (Rose 2002; Van Hagen et al. 2012) few advances were obtained in the treatment of glioblastoma. Unfortunately, GBM is associated with very poor survival due to a high resistance to radiochemotherapy (Thuy et al. 2015) including cisplatin. Therefore, the development of novel therapeutics agents able to inhibit the growth and kill glioblastoma cancer cells is urgent needed (Tanaka et al. 2013). Due to the demonstrated properties of ruthenium-based complexes and its broad range of activity against metatastic tumors, these metalodrugs emerge as an alternative for the treatment of GBM tumors. Additionally, organometallic Ru compounds have been shown to be radiosensitisers of human cancers cells (Brabec and Novakova 2006; Carter et al. 2016) and Ru isotopes have been pointed as new potential radionuclides candidates for therapy (Neves et al. 2005).

Radionuclide therapy (RNT) is rapidly growing field in nuclear medicine and can be an alternative way to decrease the toxicity and improve the specificity of antitumoral drugs. Radionuclides are employed to deliver cytotoxic dose of radiation to diseased cells/tissues (Kumar et al. 2016). We hypothesized that combination of the known properties of ruthenium complexes with the radiation produced by its neutron activation could improve the antitumor activity of these compounds offering more options for the use of ruthenium metallodrugs. To the best of our knowledge, this is the first report describing the use of neutron activation strategy to generate radioactive ruthenium complexes and to improve the activity against tumor cells. In this work, we investigated the effect of phosphine-ruthenium complexes before and after neutron activation against GBM cell lines.

## Materials and methods

### Ruthenium compounds

**Fig. 1:**
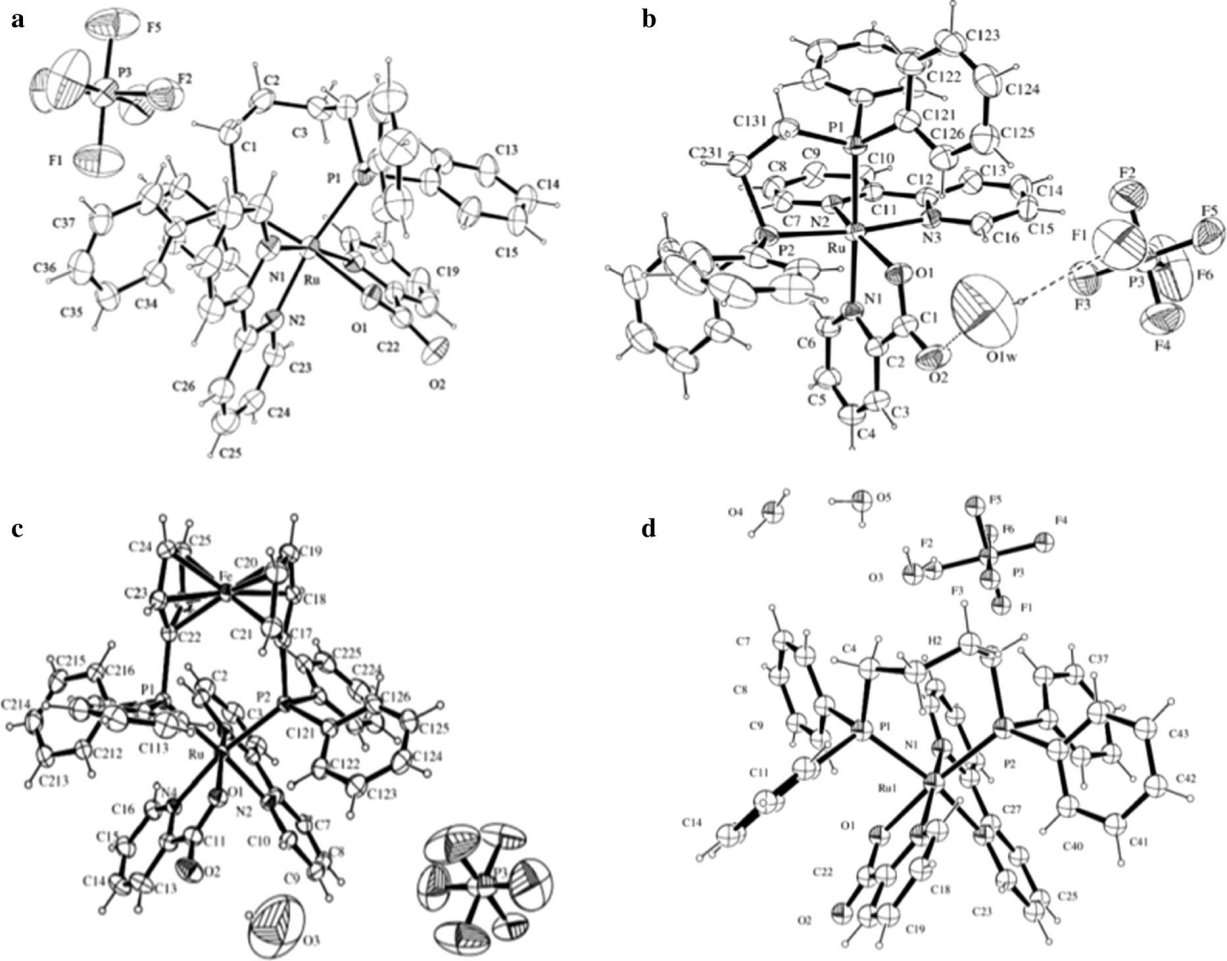
3D molecular structures of ruthenium complexes **a** Rudppb^a^, **b** Rudppe^b^, **c** Rudppf^b^ e **d** Rudppp, generated using the program ORTEP (*Oak ridge ellipsoid plot*) (^a, b^Pavan et al. 2010, 2011)

The compounds of ruthenium(II): picolinate 2,2′-Bipyridine 1,4-bis(diphenylphosphino)butane hexafluorophosphate; [Ru(pic)(bipy)(dppb)]PF6 **(1)**, 1,2-bis(diphenylphosphino)ethane; [Ru(pic)(bipy)(dppe)]PF6 **(2)**, 1,1′- bis(diphenylphosphino)ferrocene; [Ru(pic)(bipy)(dppf)]PF6 **(3)** e 1,3- bis(diphenylphosphino)propane; [Ru(pic)(bipy)(dppp)]PF6 **(4)** were synthesized at São Carlos Federal University, São Carlos (SP/Brazil) and details was published elsewhere (Dos Santos et al. 2013) and the structures are shown in Fig. 1.

Stock solutions of 0.02 M of all compounds were freshly prepared in dimethyl suphoxide (DMSO). Final use concentration was adjusted by dilutions keeping the DMSO concentration <0.5% in the experiments.

### Neutron activation of rutheniun phosphine compounds

Samples of the compounds **1**, **2, 3** and **4** were irradiated for 8 h into polyethylene flasks carried out on the Central position of the TRIGA (Training, Research, Isotopes, General Atomics) MARK-I IPR-R1 nuclear reactor at the Centro de Desenvolvimento da Tecnologia Nuclear - Comissão Nacional de Energia Nuclear (CDTN-CNEN, Belo Horizonte, Brazil), with a thermal neutronic flux of 4.1 × 10^12^ n cm^−2^ s^−1^. After neutron irradiation, samples were submitted to gamma spectrometry in a system with an HPGe detector CANBERRA 5019 and Gennie 2000 v2.0, CANBERRA software and a spectrometer Wizard 2480 in order to characterize the spectra and to determine the induced activity. The ruthenium complexes **1**, **2, 3** and **4** reached a specific activity in the range of 1.32–1.55 Gbq/mol.

### Cells lines and culture conditions

Malignant human Glioblastoma tumor cell lines U87 (p53 wild-type), T98 (p53 mutant) and normal cells MRC5 (Human Fetal Lung Fibroblast) were obtained from the American Type Culture Collection (ATCC, USA). The cells were maintained in Dulbecco’s Modified Eagle’s Medium (DMEM, Gibco), supplemented with 10% fetal bovine serum (FBS-Cultilab) and antibiotics (50 U/ml penicillin, 50 µM streptomycin), at 37 °C in a humidified 5% CO_2_ atmosphere. For all experiments, cells were seeded at appropriate concentrations to ensure exponential growth.

### Cytotoxic activity assay

The cytotoxicity of ruthenium(II) compounds was determined by measuring mitochondrial activity as a indicator of cell viability dependent on the reduction of MTT 3-(4,5-dimethylthiazol-2-yl)-2,5-diphenyl tetrazolium bromide to formazan (Mosmann 1983). Cells (1.5 × 10^3^/well) were seeded in 200 μl complete medium into 96-well microplates in triplicates. The plates were incubated at 37 °C in 5% CO_2_ for 24 h to allow cell adhesion, prior to drug testing. All radioactive or non-radioactive tested compounds were dissolved in sterile DMSO and diluted to reach final concentrations ranging from 1 × 10^−10^ to 1 × 10^−4^ mol l^−1^. Cells were exposed to the compounds for a 24 h period and then, 0.5 mg ml^−1^ of MTT solution was added to each well and further incubated for 4 h. The precipitated formazan crystals were dissolved in DMSO and the absorbance of produced color measured spectrophotometrically at 570 nm. The results were expressed as % viability of non-treated cell (control) versus drug concentration and the concentration that reduces cell viability by 50% (IC_50_) was calculated.

### Morphological analysis of treated cells

After 24 h of treatment with different ruthenium complexes at a final concentration of 1 × 10^−5^ mol l^−1^ cells were washed once with buffered phosphate saline (PBS) followed by ice cold methanol fixation for 20 min. Cells were washed again with PBS and stained with 400 ng ml^−1^ DAPI 4′,6-diamidine-2-phenylindole dihydrochloride (Sigma-Aldrich) solution. After additional washes in PBS cell nuclei were visualized on an inverted fluorescent microscopy (Nikon: 385–410 nm) and photographs were taken.

### Acridine orange-ethidium bromide (AO/EB) doubling staining assay

Cells were treated with radioactive or non-radioactive compounds **1**, **2**, **3** and **4**. After 24 h incubation at 37 °C in 5% CO_2_ atmosphere, cells were washed with PBS and further incubated for 15 min in complete medium supplemented with acridine orange (Sigma) 1 µg ml^−1^ and ethidium bromide (Sigma) 1 µg ml^−1^. Medium was removed and cells were examined under an inverted fluorescent microscope (Nikon: 530–560 nm). Photographs were taken with a Nikon camera adapted to the microscope. Acridine orange penetrates both living and dead cells emitting green fluorescence, when intercalated into normal double-stranded DNA, and red fluorescence, when bound with damaged single stranded DNA. Ethidium bromide penetrates only dead cells with damaged membranes emitting red fluorescence. Four cell types could be identified on the basis of fluorescence emission and morphological aspect of chromatin condensation and stained nuclei: (1) viable cells with uniformly bright green nuclei fluorescence and an organized structure (control); (2) early apoptotic cells with irregular green nuclei with condensed chromatin and with apoptotic bodies stained in red; (3) late apoptotic cells with orange to red nuclei with highly fragmented chromatin; (4) uniformly orange to red nuclei with an organized structure related to necrotic cells.

### Measurement of reactive oxygen species (ROS) generation

To evaluate the generation of reactive oxygen species induced by ruthenium complexes, ROS accumulation was detected using 2′,7′-dichlorodihydrofluorescein diacetate DCFH-DA (Sigma-Aldrich). DCFH-DA is a non-fluorescent compound which upon taken up by passive diffusion into cells is hydrolyzed by esterases to yeld non-permeable DCFH. In the presence of ROS, DCFH is oxidized to the fluorescent DCF (Thannickal and Fanburg 2000). Cells were treated with 1 × 10^−5^ mol l^−1^ of ruthenium complexes for 24 h and then incubated with 1 × 10^−5^ mol l^−1^ DCFH-DA for 30 min at 37 °C. Following incubation, the cells were washed twice with PBS and examined under the fluorescent microscope Nikon coupled with a digital camera at excitation and emission wavelengths of 488 and 525 nm, respectively. Quantitative analysis of fluorescence was performed using ImageJ software (ImageJ, National Institute of health, Bethesda, MD, USA) and expressed as percentages of DCF-DA fluorescence of the corresponding control.

### Statistical analysis

Data were expressed as mean ± standard deviation (SD) of three independent experiments performed in triplicates. One-tailed unpaired Student`s *t* test was used for significance testing using a p value of 0.05. Prisma Graph Pad version 5.01(Graphpad Software Inc., La Jolla, CA) was used for the statistical analysis.

## Results

### Cytotoxic activity against malignant glioma cells

All ruthenium(II)/phosphine/diimine compounds evaluated showed strong antiproliferative and cytotoxic activity on glioma cells. Compounds [Ru(pic)(bipy)(dppf)]PF6 and [Ru(pic)(bipy)(dppp)]PF6 were the most active with IC_50_ values of 3.98 ± 0.85 μM; 4.92 ± 2.46 μM for U87 cells and 3.05 ± 0.72 μM; 1.49 ± 0.50 μM for T98 cells respectively (Table 1). All the compounds tested for both U87 and T98 cell lines, except [Ru(pic)(bipy)(dppp)]PF6, showed smaller IC_50_ values than that determined for the normal control cell line MRC5. Upon neutron activation of ruthenium complexes the cytotoxicity greatly increased corresponding to IC_50_ values around 10,000 fold smaller (Table 2).

**Table 1:** Cytotoxicity of non-radioactive ruthenium complexes against glioma cells

Non-radioactives compounds	IC_50_ (µM)		
	U87	T98	MRC5
[Ru(pic)(bipy)(dppb)]PF6 (**1**)	16.1 ± 3.3	11.9 ± 5.9	41.0 ± 10.1
[Ru(pic)(bipy)(dppe)]PF6 (**2**)	27.8 ± 11.7	19.0 ± 5.9	82.1 ± 9.1
[Ru(pic)(bipy)(dppf)]PF6 (**3**)	4.0 ± 8.5	3.0 ± 0.7	8.9 ± 2.3
[Ru(pic)(bipy)(dppp)]PF6 (**4**)	4.9 ± 2.5	1.5 ± 0.5	1.47 ± 0.6
Cisplatin	1.8 ± 0.2	5.3 ± 1.9	5.05 ± 0.7

**Table 2:** Cytotoxicity of neutron activated ruthenium complexes against glioma cells

Radioactive compounds	IC_50_ (µM)		
	U87	T98	MRC5
[^103^Ru(pic)(bipy)(dppb)]PF6 (**1**)	2.4 × 10^−4^ ± 1.3 × 10^−4^	3.4 × 10^−4^ ± 9.4 × 10^−5^	3.9 × 10^−4^ ± 1.0 × 10^−4^
[^103^Ru(pic)(bipy)(dppe)]PF6 (**2**)	2.3 × 10^−4^ ± 1.5 × 10^−4^	5.2 × 10^−4^ ± 9.4 × 10^−5^	2.0 × 10^−4^ ± 5.5 × 10^−5^
[^103^Ru(pic)(bipy)(dppf)]PF6 (**3**)	4.2 × 10^−4^ ± 1.7 × 10^−4^	5.4 × 10^−4^ ± 2.3 × 10^−5^	3.8 × 10^−4^ ± 4.1 × 10^−5^
[^103^Ru(pic)(bipy)(dppp)]PF6 (**4**)	3.1 × 10^−4^ ± 1.7 × 10^−5^	6.2 × 10^−4^ ± 8.7 × 10^−5^	4.7 × 10^−4^ ± 0

Ruthenium complexes presented selectivity index (SI = IC_50_ MRC5/IC_50_ tumor cell) ranging from 0.29 to 4.32. These values were comparable to that determined for cisplatin in U87 cells. However, Selectivity indexes were higher and better than cisplatin for T98 tumor cell line harboring the tumor suppressor TP53 mutant gene. [Ru(pic)(bipy)(dppe)]PF6 was the compound that showed the highest SI among the ruthenium compound tested (Table 3).

**Table 3:** Selectivity index of non-radioactive and neutron activated ruthenium complexes in glioma cells

Selectivity index				
Compounds	Non-radioactive		Radioactive	
	U87	T98	U87	T98
[Ru(pic)(bipy)(dppb)]PF6 (**1**)	2.55	3.45	1.63	1.14
[Ru(pic)(bipy)(dppe)]PF6 (**2**)	2.95	4.32	0.87	0.39
[Ru(pic)(bipy)(dppf)]PF6 (**3**)	2.23	2.91	0.90	0.70
[Ru(pic)(bipy)(dppp)]PF6 (**4**)	0.29	0.98	1.52	0.76
Cisplatin	2.87	0.95	–	–

### Morphological analysis of tumor cells treated with unmodified and neutron activated ruthenium complexes

**Fig. 2:**
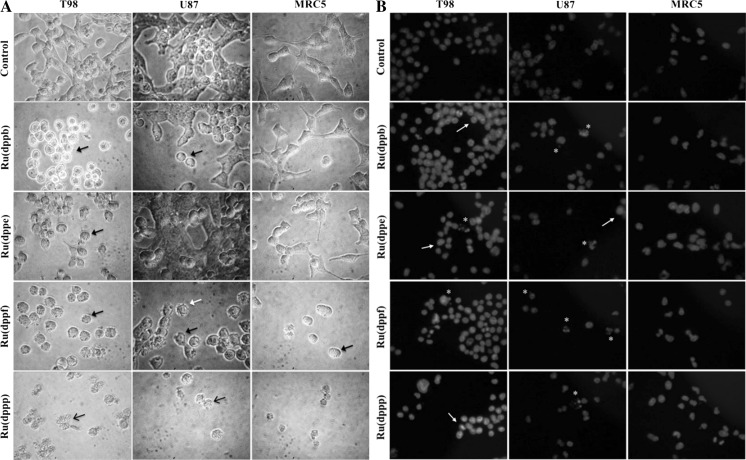
Effect of non-radioactive ruthenium(II) complexes **1**, **2**, **3** and **4** on the morphology of T98, U87 and MRC5 cell lines. **a** After 24 h treatment, morphological changes such as cell rounding and shrinkage (*black closed arrow*), irregularities on cell membrane (*black open arrow*) and blebs formation (*white arrow*) can be observed under phase contrast. **b** Cells were treated with were stained with DAPI. Nuclear condensation (*arrows*), nuclear fragmentation (*white asterisks*) and formation of apoptotic bodies (*yellow asterisks*) were seen in cells treated with these compounds Concentration: 10 µM. Amplification: ×400

**Fig. 3:**
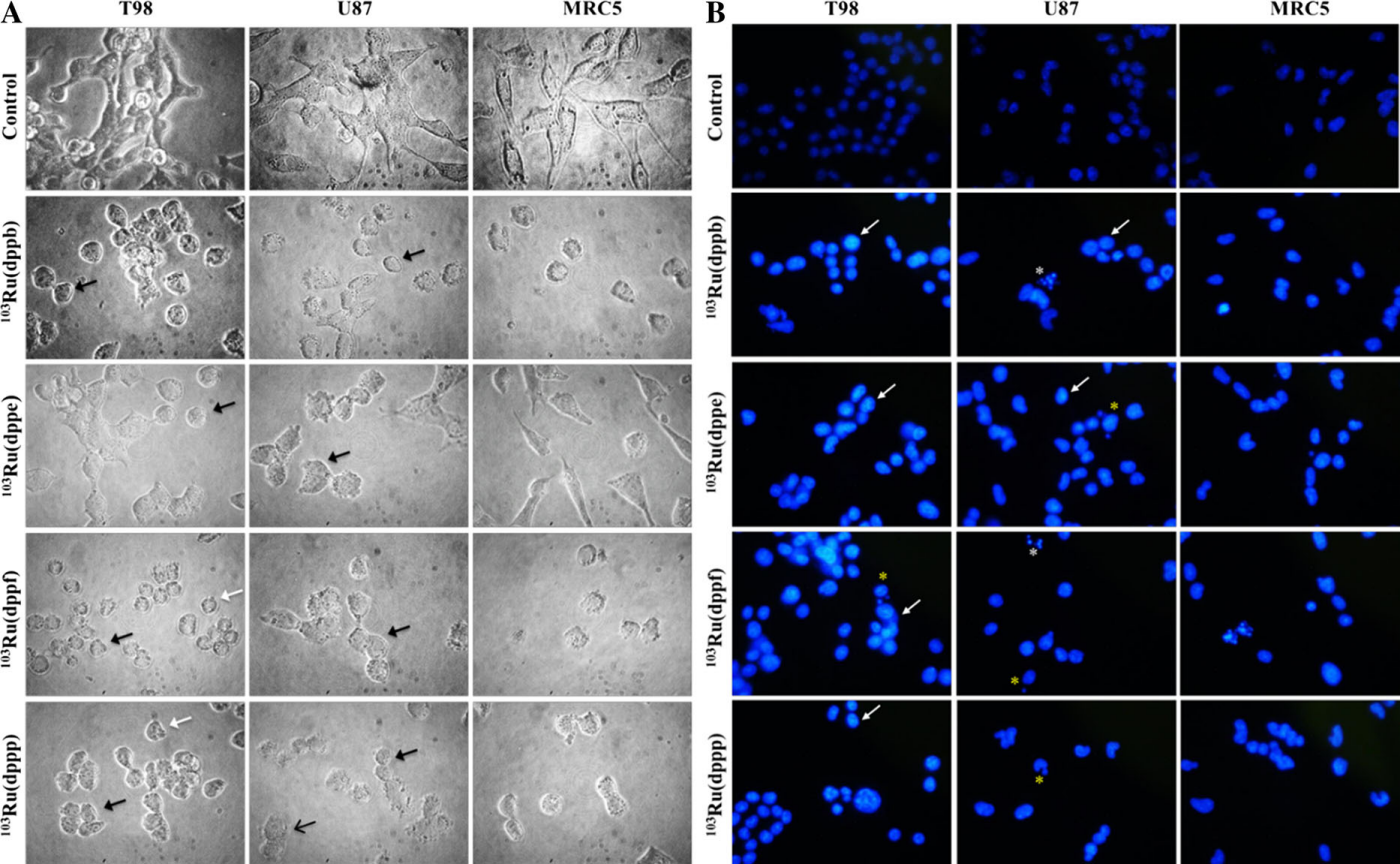
T98, U87 and MRC5 cells treated with neutron activated ruthenium(II) complexes **1**, **2**, **3** and **4**. **a** After 24 h treatment, morphological changes such as cell rounding and shrinkage (*black closed arrow*), irregularities in membrane (*black open arrow*) and blebs formation (*white arrow*) could be observed under phase contrast microscope. **b** Cells treated were stained with DAPI and nuclei examined under fluorescent microscope. Nuclear condensation (*arrows*), nuclear fragmentation (*white asterisks*) and formation of apoptotic bodies (*yellow asterisks*) were seen in cells treated with these compounds. Concentration: 10 µM. Amplification: ×400

All ruthenium(II)/phosphine/diimine compounds tested induced morphological alterations on glioma cell lines independent of the p53 protein status. Morphological and culture behavior alterations such as cytoplasmic and cell membrane retraction changing the spindle-like cell shape, commonly seen in normal cultured glial derived cells, leading to a transition from the characteristic fusiform to rounded shape cells, detachment from the plate culture, cell shrinkage and membrane blebs formation. After DAPI staining, chromatin condensation, nuclear fragmentation and formation of apoptotic bodies could be also observed (Fig. 2). Neutron activated compounds also induced the same morphological alterations at lower concentrations (Fig. 3).

### Determination of the type of cell death induced by ruthenium complexes using acridine-orange/ethidium bromide (AO/EB) double staining

**Fig. 4:**
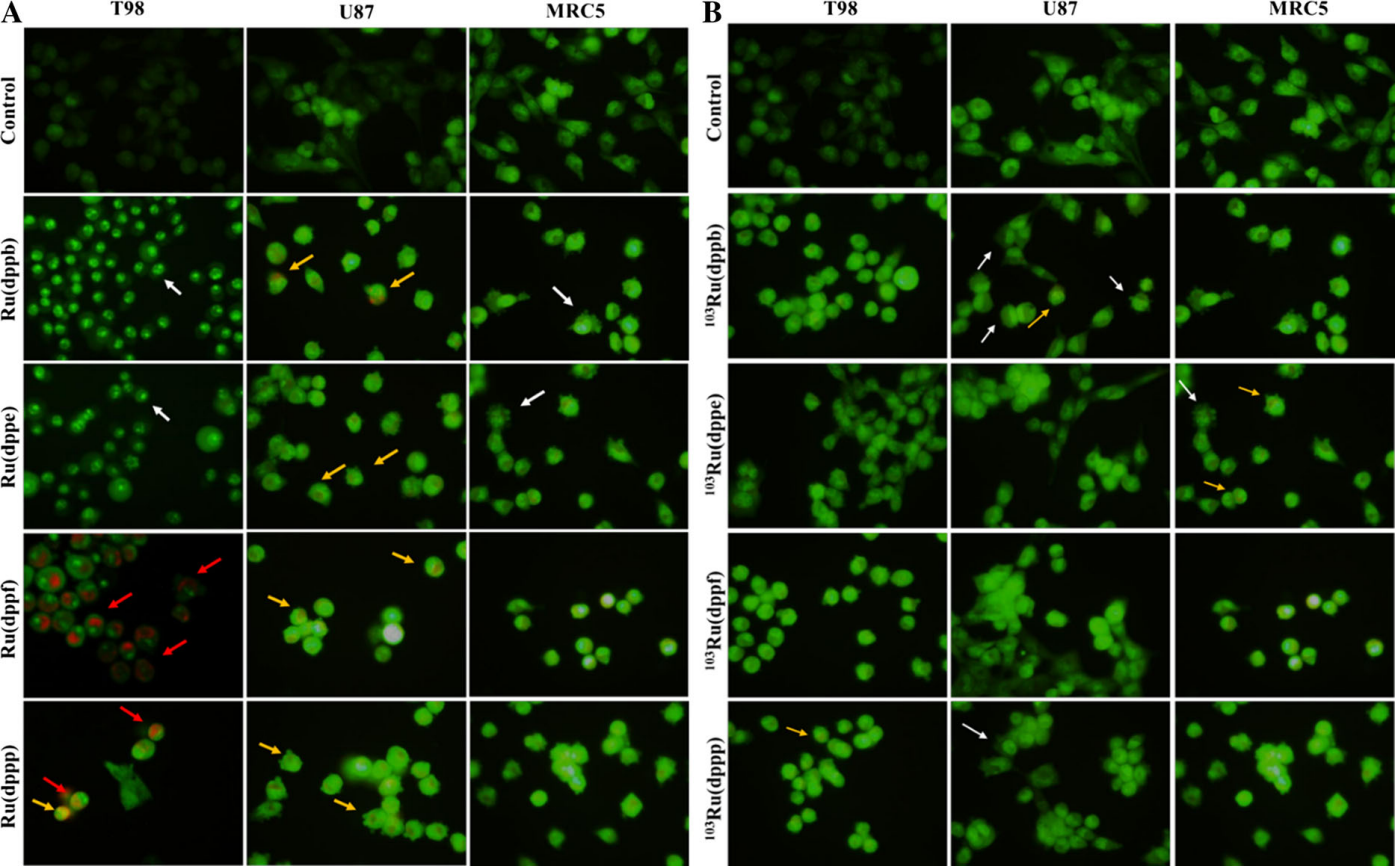
Detection of cell death induced by ruthenium complexes. Cells T98, U87 and MRC5 were treated with ruthenium complexes (10 µM) for 24 h followed by double staining with acridina orange/ethidium bromide. Evidence of acid autophagossomes (*yellow arrow*), apoptosis (*white arrow*) and some necrotic cells (red arrows). **a** Treatment non-radioactive. **b** Treatment with neutron activated compounds. Amplification: ×400

In order to correlate the observed morphological alterations with decreased cell viability and to identify early/late apoptotic events, necrose and autophagy, we double stained cells with acridine orange (AO) and ethidium bromide (EB) after 24 h treatment. Viable cells could be identified by bright homogeneous green nuclei in untreated cells (AO). Cells presenting early apoptotic events were identified by irregularly structured green nuclei with condensed chromatin and orange or light red patches. Late apoptotic cells contained positively stained nuclei with both dyes (EB and AO) appearing orange or light red with the apoptotic bodies. Nuclei of necrotic cells had intact chromatin and appeared red. In control cells, all nuclei appeared green with a regular spherical or oval structure and chromatin organization (Fig. 4).

### Measurement of reactive oxygen species

**Fig. 5:**
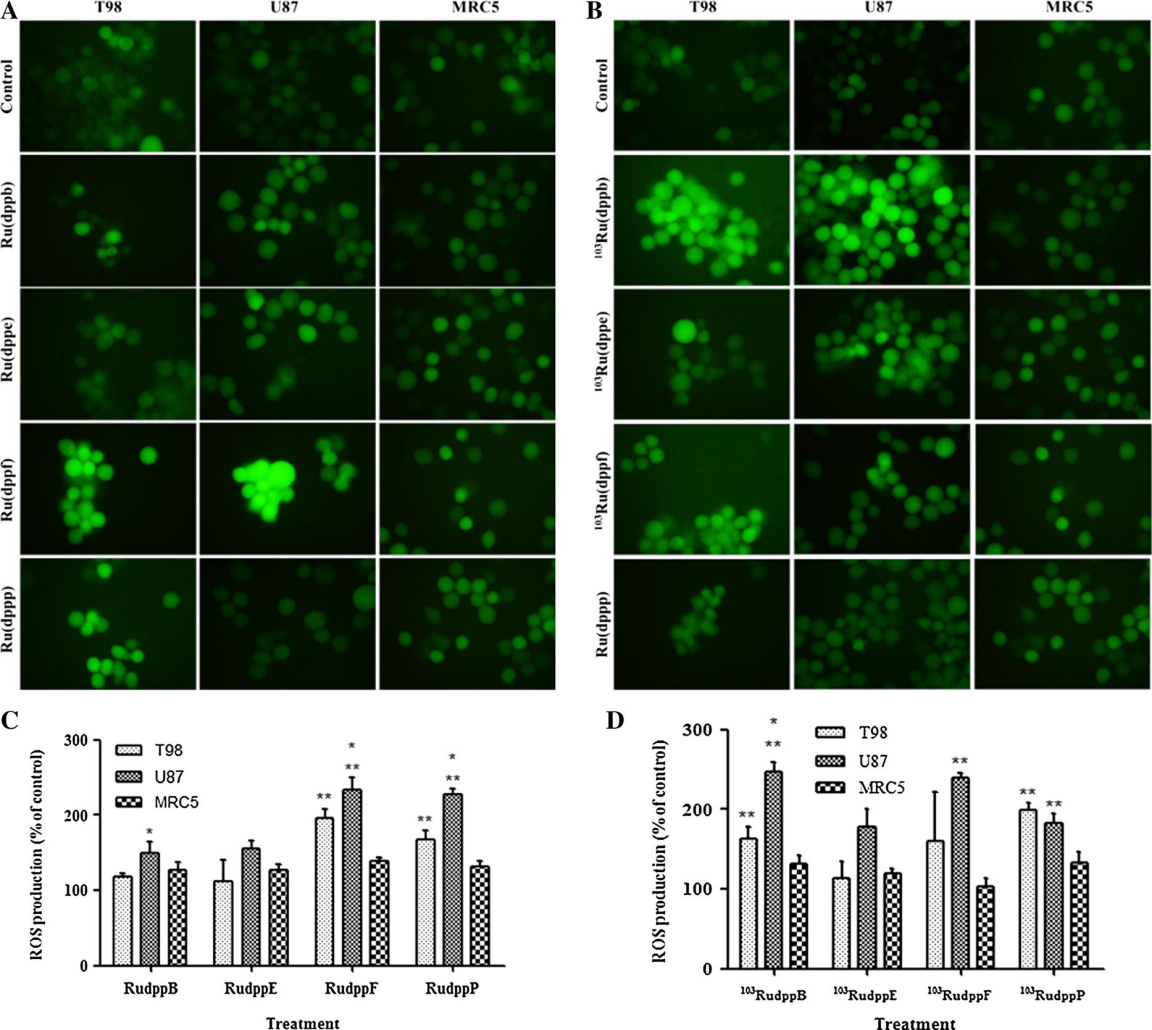
Generation of reactive oxygen species (ROS) in cells treated with compounds **1**, **2**, **3** and **4**. After 24 h treatment cells were stained with DCF-DA. Treated glioma cells show increased fluorescence intensity (amplification ×400). **a** Non-radioactive treatment. **b** Radioactive Treatment. C and D show ROS production (measured as relative fluorescence increase) in cells treated with compounds **1**, **2**, **3** and **4**. Concentration: (10 µM). ^*^Statistically significant difference from T98; ^**^statistically significant difference from MRC5

Generation of reactive oxygen species was determined by detection and measurement of fluorescence produced by the oxidation of the non-fluorescent 2′,7′-dichlorodihydrofluorescein-diacetate (DCFH-DA) into the fluorescent 2′,7′-dichlorodihydrofluorescein (DCF) by free radicals inside the cell. As shown in Fig. 4, treatment of glioma cells with ruthenium complexes for 24 h resulted in marked increase of the intracellular ROS levels (Fig. 5). These levels were higher in tumor cells than in the control MRC5 cells depending on the compound analyzed.

## Discussion

New ruthenium(II)/phosphine/diimine compounds have been synthesized and their biological activities evaluated. Similar to other ruthenium complexes, these new compounds have demonstrated promising activity on glioblastoma tumor cells bearing different genetic backgrounds. Furthermore the compounds tested here showed better activity than the well-known cisplatin drug. Ruthenium based compounds showed IC_50_ in the range of micromolar concentrations. However, upon neutron activation we could observe a decrease of approximately 10,000 fold in the IC_50_ values demonstrating that this approach largely increased the toxicity of the primary compound and revealing a great potential for neutron activation of ruthenium based metallodrugs as a strategy to increase cytotoxicity of these compounds. To our knowledge this is the first report showing the use of this approach to try to increase the cytotoxicity of ruthenium compounds. Even considering the fact that the increased cytotoxicity affected the normal cell line used MRC5, still this strategy may be useful after some modifications targeting ruthenium complexes to a more specific site into cancer cells.

Some of the advantages of using ions of transition metals such as ruthenium instead platinum include the availability of additional coordination sites in octahedral complexes and altered shape of the complex. In addition, the changes in oxidation state, alterations in ligand affinity, substitution kinetics and potential for photodynamic approaches to therapy turn these complexes theoretically attractive. Considering the octahedral structure of ruthenium(II) complexes as opposed to the square-planar geometry of platinum(II) compounds, ruthenium(II) anticancer complexes probably function in a way different from that of cisplatin. Our results suggested that in addition to the known DNA affinity of ruthenium complexes, the generation of reactive oxygen species may be other mechanism by which ruthenium can induce cell death. In fact, ROS production is associated in many forms of apoptosis through mediation of signaling transduction processes (Suzuki et al. 1997). We showed that neutron activated ruthenium compounds were able to generate more ROS than their counterparts which correlated well with their increased cytotoxicity.

Employment of radiotherapeutical agents has always been an important strategy to treat cancer patients. Furthermore, the use of molecular carriers that may provide vehicles for selective deposition of radioactivity in the tumor region has received great interest in the past decades. Ruthenium isotopes decay by electron capture and emit low energy electrons consisting mainly of Auger and Coster–Kronig electrons. It is well-known that incorporation of Auger–electron emmiters within the cell nucleus produce high radiotoxicity (Volkert et al. 1991). This is due to the deposition of a concentrated amount of energy emitted in the form of a shower of Auger and Coster-Kronig electrons with energies ranging from a few to several hundred electron volts into an extremely small volume within the nuclear DNA (Makrigiorgos et al. 1990). The higher cytotoxicity showed in this work after neutron activation of ruthenium compounds may be due to this mechanism. In order to be used in radionuclide therapy methods of selective guidance to target cells and subsequent introduction of these radionuclides with good selectivity into the nucleus must be found. Ruthenium complexes have shown to have high affinity to DNA which could be a way to target the radionuclide to the nucleus. In fact, ^106^Ru has been reported as a good strategy in brachytherapy to treat uveal melanoma (Barker et al. 2014; Naseripour et al. 2016). Nevertheless, further studies are necessary to better evaluate the potential of neutron activated ruthenium compounds in radionuclide therapy for glioblastomas.

## Conclusions

Phosphine-ruthenium-based complexes decrease glioblastoma cell proliferation and show IC_50_ in the range of micromolar concentrations. Neutron activation of these compounds is able to increase their cytotoxicity and may be a good strategy to radionuclide therapy.
